# Determinants of emergency department disposition of patients with traumatic brain injury in Uganda: results from a registry

**DOI:** 10.1136/tsaco-2018-000253

**Published:** 2018-12-19

**Authors:** Amber Mehmood, Nukhba Zia, Olive Kobusingye, Rukia H Namaganda, Hussein Ssenyonjo, Joel Kiryabwire, Adnan A Hyder

**Affiliations:** 1 Johns Hopkins International Injury Research Unit, Health Systems Program, Department of International Health, Johns Hopkins University Bloomberg School of Public Health, Baltimore, Maryland, USA; 2 Makerere University’s School of Public Health, Kampala, Uganda; 3 Department of Neurosurgery, Mulago Hospital, Kampala, Uganda; 4 Global Health, George Washington University Milken Institute School of Public Health, Washington, DC, USA

**Keywords:** traumatic brain injury, glasgow coma scale, head injury, uganda, kampala trauma score, revised trauma score, outcomes

## Abstract

**Background:**

Traumatic brain injuries (TBIs) are a common cause of emergency department (ED) visits and hospital admissions in Kampala, Uganda. The objective of this study was to assess determinants of ED discharge disposition based on patient demographic and injury characteristics. Four ED outcomes were considered: discharge home, hospital admission, death, and others.

**Methods:**

This prospective study was conducted at Mulago National Referral Hospital, Kampala, Uganda, from May 2016 to July 2017. Patients of all age groups presenting with TBI were included. Patient demographics, external causes of injury, TBI characteristics, and disposition from EDs were noted. Injury severity was estimated using the Glasgow Coma Scale (GCS), Kampala Trauma Score (KTS), and the Revised Trauma Score (RTS). A multinomial logistic regression model was used to estimate conditional ORs of hospital admission, death, and other dispositions compared with the reference category “discharged home”.

**Results:**

A total of 3944 patients were included in the study with a male versus female ratio of 5.5:1 and a mean age of 28.5 years (SD=14.2). Patients had closed head injuries in 62.9% of cases. The leading causes of TBIs were road traffic crashes (58.8%) and intentional injuries (28.7%). There was no significant difference between the four discharge categories with respect to age, sex, mode of arrival, cause of TBI, place of injury, type of head injury, transport time, and RTS (p>0.05). There were statistically significant differences between the four discharge categories for a number of serious injuries, GCS on arrival, change in GCS, and KTS. In a multinomial logistic regression model, change in GCS, area of residence, number of serious injuries, and KTS were significant predictors of ED disposition.

**Discussion:**

This study provides evidence that ED disposition of patients with TBI is differentially affected by injury characteristics and is largely dependent on injury severity and change in GCS during ED stay.

**Level of evidence:**

Level II.

## Introduction

Traumatic brain injury (TBI) is dubbed as a silent epidemic affecting an estimated 69 million individuals each year.^1 2^ TBI is also among the leading causes of brain disorders and disability worldwide. Road traffic injuries (RTIs) account for nearly 60% of TBI cases and are ranked as the eighth leading cause of death in 2015, accounting for 1.2 million deaths globally, an increase of about 19.7% since 1990.^3^ Other important contributors to TBIs include falls (20%–30%), violence (10%), and occupational or sports-related injuries (10%).^2 3^

TBI poses a large burden on healthcare systems in both low-income and high-income countries, especially at the extremes of age.^1 4^ According to new estimates, approximately 50 million people from low-income and middle-income countries (LMICs) sustain a TBI annually, with an incidence of 811 per 100 000 population.^1^ TBI incidence in sub-Saharan Africa was previously reported to range between 150 and 170 per 100 000, but new estimates show that the annual incidence could be as high as 801 per 100 000 (95% CI 732 to 871).^1 2^ This is despite under-reporting due to poor surveillance and difficulty in accessing care.^2^ Rapid motorization and regional conflict have been named as important causes of higher TBI incidence in the region.^5^

Countries like Uganda suffer a disproportionate burden of TBIs, with higher mortality rates and both short-term and long-term poor outcomes. The number of deaths from RTI in Uganda has seen a 154.9% increase in a 15-year period, from 3059 in 1990 to nearly 7797 in 2015.^3^ Population growth and unregulated modernization have also resulted in an increased incidence of TBIs in the country.^6–8^ For instance, a study from Kampala estimated an annual all-injury incidence of 116 per 1000, with an injury mortality rate of 220 per 100 000 population.^9^ The leading causes of injuries in this study were RTIs, followed by firearms. Another study estimated that the cumulative incidence of TBI hospital admission in Uganda is 89 per 100 000 population.^5^

Much like other injury victims, patient characteristics, injury severity, and access to healthcare also influence outcomes in patients with TBI.^10–12^ In-hospital survival is correspondingly impacted by a number of factors including availability of services, implementation of management guidelines, and quality of care.^13 14^ Some facility-based studies from Uganda have highlighted a range of issues including a lack of resources and standardized care from across emergency departments (EDs) to intensive care units (ICUs), with hospital-based injury mortality ranging between 45.3% and 75%.^15–17^ Despite these alarming figures, the lack of national and disaggregated data on TBI does not allow for a complete understanding of the magnitude of the TBI burden in Uganda and poses a barrier to defining risks, identifying vulnerable groups, and assessing the impact of potential TBI interventions.

To better address the growing burden of TBIs, there are a number of critical gaps in knowledge that need to be tackled.^5^ The WHO has recognized the paucity of good evidence in the existing literature on TBI care and management especially from LMICs, and has outlined a number of research priorities for TBIs, including identification of specific and modifiable risk factors (such as alcohol or drug use or environmental risks), understanding the effects of other injuries, and determining factors associated with poor prognosis in mild TBIs.^18^ The lack of hospital-based data on the burden of TBIs, injury characteristics, and quality of care has resulted in slow progress toward meeting the health needs of the most vulnerable populations, namely comprehensive programs aimed at prevention and care.

This study was conducted to understand the burden, risk factors, and outcome of TBI for those patients who present at a tertiary-care hospital in Kampala, Uganda. The specific aims of the study are to (1) describe the burden and characteristics of patients presenting with TBI in a large tertiary-care hospital of Kampala, (2) describe injury circumstances and measures of injury severity, and (3) identify factors causing a significant impact on the disposition of ED patients with TBI. This study aims to generate relevant information to guide the best practices and policies for TBI management in Uganda and other low-resource settings.

## Methods

This study is based on data from the “Kampala internet-based Traumatic Brain Injury Registry (KiTBIR)” implemented at the Mulago National Referral Hospital, Kampala, Uganda.^19^ Mulago National Referral Hospital, commonly referred to as Mulago Hospital, is the largest tertiary-care hospital in Uganda, located in the capital city Kampala. It is also the teaching hospital of Makerere University and one of the two national referral hospitals. This 1500-bed government hospital offers specialist services in various surgical disciplines and is the only tertiary-care neurosurgical center in the country. It admits around 140 000 patients per year and employs four of the country’s five neurosurgeons.

KiTBIR was launched in 2016 using a mobile health platform, developed and customized specifically for Uganda as a collaborative effort between Johns Hopkins Bloomberg School of Public Health, Makerere University and Mulago Hospital. The purpose of this registry was to describe the TBI burden and document in-hospital TBI care throughout the course of hospital stay. Hence, data were collected on patients’ demographics, circumstances of injury, TBI causes, prehospital care, mode of arrival and approximate duration between injury and arrival in the first medical facility, hospital assessment and care, severity as measured through the Glasgow Coma Scale (GCS), Kampala Trauma Score (KTS), and Revised Trauma Score (RTS), and patient outcomes at ED and inpatient discharge.^20^ The initial clinical assessment was recorded as soon as the patients arrived in the ED, but information on demographics, prehospital transport, injury event, and care provision was collected after stabilization and continued until discharge from the hospital. Data collection was done by the nursing staff in the ED who were trained specifically in data abstraction from medical charts, patient interview, consent procedures, and data entry and submission using a tablet-based registry application. The data on tablets were submitted via secure networks to a secured server protected by limited access and strong password systems.

Patients of all age groups and gender presenting to the ED with suspected or documented TBIs between May 2016 and July 2017 were included in this study. TBI was defined based on the history of direct injury to the head or on a mechanism suspected to cause TBIs, such as falls, RTIs, or assaults that cause injuries involving multiple body regions. These included both blunt and penetrating trauma, with or without history of loss of consciousness. Suspected cases of TBI were evaluated for eligibility based on their initial assessment in the ED. Patients with no evidence of TBI after a detailed history and physical examination, or who had other reasons to explain altered consciousness (such as those with meningitis, stroke, drugs or alcohol intake with no associated injury), were excluded from the study. Patients who did not fulfill the eligibility criteria, or those for whom consent, assent, and/or parental permission could not be obtained, were excluded.

### Data analysis

This analysis focused on data collected up to the time of ED disposition or within 24 hours of ED arrival, whichever came first. The main outcome measure, ED disposition, was divided into four categories: “Discharged home”, “Admitted in hospital”, “Died in ED”, and “Others”. “Others” included situations where patients were referred to another facility, left against medical advice (AMA), or were waiting for final decision on ED disposition even after 24 hours of ED stay. Age was categorized into three groups based on the pattern of age distribution in the data set: 0 to 18, 19 to 45, and >45 years. GCS reflecting TBI severity was converted into three ordinal categories (mild: 13–15; moderate: 9–12; severe:<9). RTS and KTS were taken as continuous variables.^21 22^

Descriptive statistics and tabulations were generated for data related to patient characteristics, prehospital details, causes of TBI, injury characteristics, and ED dispositions. Pearson’s χ^2^ test was used to assess the sample distribution between outcome categories.^23^ A simple multinomial logistic regression was carried out with <0.05 level of significance for ED disposition by patient characteristics, injury circumstances, and injury severity as measured by GCS on arrival and changes in GCS during ED stay, using “discharged home” as reference category.^24^ Multivariable multinomial logistic regression for ED disposition was conducted with all variables, checked for confounding followed by goodness-of-fit test.^24 25^ The final model was selected based on the lowest Akaike Information Criterion (AIC) demonstrating statistically significant conditional ORs (cORs) of independent predictors for ED disposition.^26^ Statistical analysis was performed using Stata SE V.15.1.

## Results

### Patient characteristics

There were 3944 patients included in the registry during the study period, majority of them (n=3339; 84.7%) were male (table 1). Most patients with TBI belonged to the 19 to 45 years age group (n=3034; 71.6%) across both sexes, with a mean age of 28.5 years±14.21. Almost half of the patients came from rural areas outside Kampala city, 45% were married, and another 45% were single. A little over half (56%) had only received primary education, and 37.4% were high school graduates or had received higher education. Approximately 35% (n=1379) of patients were discharged from the ED, whereas 43% (n=1697) were admitted for further inpatient management. Only 2.7% (n=109) of the patients died in the ED, whereas another 19% (n=759) were still waiting in the ED, some of them were eventually referred to other hospitals or left AMA. Approximately 15% of all patients were still waiting in the ED for final disposition at 24 hours. There was no difference between mean age and sex distribution among different categories of discharge disposition.

**Table 1:** Characteristics of patients with TBI and injury circumstances

	Discharged (n=1379)	Admitted (n=1697)	Died (n=109)	Others (n=759)	P value
Age, mean (SD)	28.24 (14.18)	28.85 (14.42)	28.51 (14.6)	28.19 (13.75)	0.61
Sex, n (%)					
Male (n=3339)	1155 (34.6)	1456 (43.61)	91 (2.73)	637 (19.08)	0.39
Female (n=605)	224 (37.02)	241 (39.83)	18 (2.98)	122 (20.17)	
Area of residence, n (%)					
Urban (n=1993)	653 (32.76)	891 (44.71)	70 (3.51)	379 (19.02)	0.009
Rural (n=1946)	724 (37.2)	805 (41.37)	39 (2.0)	378 (19.42)	
Unknown (n=4)	2 (50)	1 (25)	0 (0)	1 (25)	
Place of injury, n (%)					
Home (n=367)	142 (38.69)	155 (42.23)	8 (2.18)	62 (16.89)	0.75
Occupational area (n=402)	136 (33.83)	170 (42.29)	14 (3.48)	82 (20.4)	
Street /Highway (n=3026)	1046 (34.57)	1310 (43.29)	85 (2.81)	585 (19.33)	
Others (n=149)	55 (36.91)	62 (41.61)	2 (1.34)	30 (20.13)	
Mode of arrival, n (%)					
Private vehicle (n=1146)	409 (35.69)	495 (43.19)	31 (2.71)	211 (18.41)	0.21
Ambulance (n=1057)	375 (35.48)	442 (41.72)	40 (3.78)	201 (19.02)	
Police vehicle (n=1337)	465 (34.78)	576 (43.08)	35 (2.62)	261 (19.52)	
Motorcycle taxi (n=318)	99 (31.13)	151 (47.48)	2 (0.63)	66 (20.25)	
Others (n=86)	31 (36.05)	34 (39.53)	1 (1.16)	20 (23.26)	

TBI, traumatic brain injury.

### Injury characteristics and circumstances

The majority of TBIs were a result of RTIs (n=2325; 58.9%), but a quarter were caused by intentional injuries including assault and self-harm (n=1135; 28.8%); falls accounted for only 7.3% (n=289) of TBIs. Among patients with TBI, 76.7% (n=3026) of all injuries took place on streets or highways, whereas another 10.2% (n=402) occurred in occupational areas, such as trade and business, farms, or factories (table 2). The distribution of causes of injuries was comparable across discharge categories.

**Table 2:** Injury characteristics and severity of patients presenting with TBI in Mulago Hospital

	Discharged home	Admitted	Died	Others	P value
Cause of TBI					
RTI (n=2325)	781 (33.59)	1027 (44.17)	63 (2.71)	454 (19.53)	0.38
Falls (n=289)	107 (37.02)	112 (38.75)	10 (3.46)	60 (20.76)	
Intentional injuries (n=1135)	421 (37.09)	483 (42.56)	30 (2.64)	201 (17.71)	
Others (n=195)	70 (35.9)	75 (38.46)	6 (3.08)	44 (22.56)	
Type of head injury					
Open (n=1432)	492 (34.36)	629 (43.92)	45 (3.14)	266 (18.58)	0.555
Closed (n=2483)	879 (35.4)	1055 (42.49)	62 (2.5)	487 (19.61)	
Others (n=29)	8 (27.6)	13 (44.83)	2 (6.9)	6 (20.7)	
GCS on arrival					
Mean (SD)	12.33 (3.26)	12.3 (3.32)	12.44 (3.25)	12.55 (3.34)	0.356
Mild, 13–15 (n=2428)	840 (34.6)	1027 (42.3)	65 (2.68)	496 (20.43)	0.337
Moderate, 9–12 (n=871)	310 (35.59)	392 (45.01)	25 (2.87)	144 (16.53)	
Severe, <9 (645)	229 (35.5)	278 (43.1)	19 (2.95)	119 (18.45)	
Number of serious injuries (n=3941)					
None (n=800)	267 (19.38)	341 (20.12)	12 (11.0)	180 (23.72)	0.001
One (n=3033)	1079 (78.3)	1303 (76.87)	89 (81.65)	562 (74.04)	
Two or more (n=108)	32 (2.32)	51 (3.01)	8 (7.34)	17 (2.24)	
Change in GCS (n=3944)					
No change (n=1200)	491 (40.92)	439 (36.58)	6 (0.5)	264(22)	0.000
GCS dropped (n=1124)	234 (20.82)	605 (53.83)	91 (8.1)	194 (17.26)	
GCS improved (n=1620)	654 (40.37)	653 (40.31)	12 (0.74)	301 (18.58)	
Kampala Trauma Score (n-3941)	11.32 (2.14)	11.13 (2.22)	11.34 (2.44)	11.06 (2.27)	0.030
Revised Trauma Score (n=3944)	6.38 (1.57)	6.33 (1.64)	6.36 (1.56)	6.36 (1.61)	0.852

GCS, Glasgow Coma Scale; RTI, road traffic injury; TBI, traumatic brain injury.

**Figure 1:**
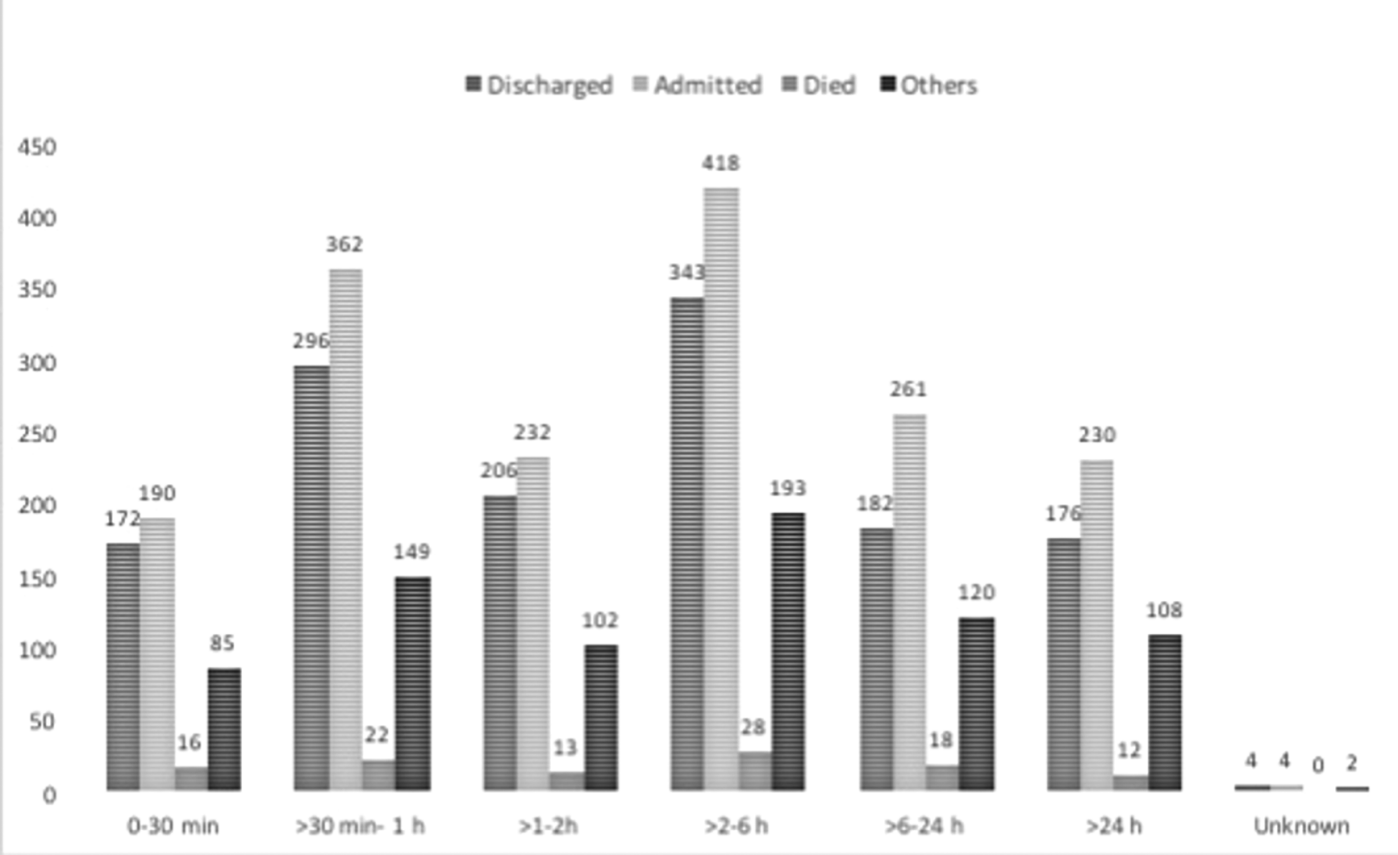
Interval between injury and arrival to the first hospital.

Only 42.1% (n=1657) of patients with TBI arrived in a hospital within the first hour of injury, whereas another 20.7% (n=819) arrived between 1 and 2 hours (figure 1). For 37% (n=1468) of patients, the duration was more than 2 hours, with 11.6% (n=460) reaching the hospital after 24 hours. There was no significant difference between transport time among different discharge categories (p=0.06). The transport time was different for different causes of TBI (p=0.00), with RTIs comprising 59% (n=987) of all TBI victims arriving within 1 hour, followed by intentional injuries (n=457; 27%) and falls (n=132; 8%). Over half (56.3%) of the patients received care in another facility prior to arrival in the Mulago Hospital.

**Figure 2:**
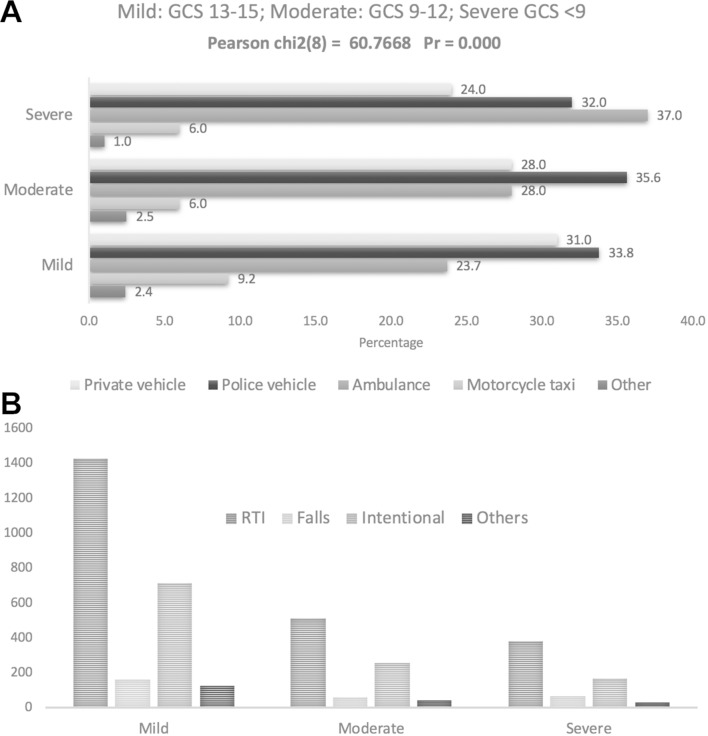
TBI severity and its association with mode TBI. (A) TBI severity and mode of arrival; (B) TBI severity vs. causes of TBI. GCS, Glasgow Coma Scale; RTI, road traffic injuries; TBI, traumatic brain injury.

The most common mode of arrival was police vehicle (n=1337; 33.9%), followed by private vehicle (n=1146; 29%) and ambulance (n=1057; 26.8%). Approximately 8% (n=318) of patients reached the hospital by motorcycle taxi. Statistically significant differences between different modes of arrival and TBI severity were observed (figure 2A). Although police vehicles were the most common mode of transport for all patients with TBI, the majority of severe TBIs were brought in by ambulance. Private vehicles were commonly used for patients with mild to moderately severe TBI (p=0.00).

The majority of patients had a closed head injury (n=2483; 62.9%). Compared with other causes of TBI, RTIs were significantly associated (p=0.04) with severe head injuries (figure 2B). The majority (n=3033; 76.9%) of patients had only one serious injury, which was defined as an injury potentially warranting hospital admission. Only 3% had two or more injuries, although this proportion was significantly higher among those who died in the ED, versus those who were discharged or admitted (p=0.001).

Fifty percent of patients had GCS ≥14 with an IQR of 5, although the overall mean GCS was 12.9±3.1 on arrival. The distribution of GCS categories (p=0.35), sex (p=0.36), and age categories (p=0.24) was comparable among different ED dispositions. Over two-thirds of patients (n=2428; 69.57%) had been categorized as having mild head injuries. The mean KTS was 11.19±2.21 and the mean RTS was 6.35±1.61.

Postresuscitation GCS was also recorded for all patients, and three categories were identified: those whose GCS remained unchanged (n=1200; 30.4%), those whose GCS dropped by at least one point (n=1124; 28.4%), and those whose GCS improved at least by one point or more after resuscitation (n=1620; 41.1%). Most patients who were discharged from the ED demonstrated an unchanged or improved GCS (n=1145; 83%), those who were admitted had an unchanged or dropped GCS (n=1697; 61.5%), whereas the majority of those who died (n=91; 83.4%) had a documented drop in GCS (table 2).

### Multinomial logistic regression

A multinomial logistic regression model was used to identify factors associated with the disposition of a patient with TBI. Table 3 shows the unadjusted and adjusted cOR for each outcome category when compared with the reference category “discharged home”. In the unadjusted model, each predictor variable’s odds were compared with the reference category, independent of other predictors. Being from rural areas had a significantly lower cOR of death, although this finding was not consistent across other discharge categories. A drop in GCS had significantly higher conditional odds for admission (cOR 2.89; 95% CI 2.37 to 3.52), death (cOR 31.82; 95% CI 13.72 to 73.76), or others (cOR 1.54 95% CI 1.21 to 1.96) as compared with discharge home. A higher KTS had statistically significantly lower conditional odds for admission (cOR 0.96; 95% CI 0.93 to 0.99) or others (cOR 0.94; 95% CI 0.91 to 0.98) when compared with the reference. Similarly, GCS of 9 to 12 had significantly lower odds for being in the “others” category, when compared with the patients with mild TBI. Having two or more serious injuries placed the patients in higher conditional odds (cOR 5.96; 95% CI 1.13 to 3.45 for death), whereas having only one serious injury resulted in statistically significantly lower odds of being in the “others” category (cOR 0.77; 95% CI 0.62 to 0.95). Sex, age, place of injury, mode of arrival, transport time, cause or type of TBI, and RTS were not statistically associated with ED disposition.

**Table 3:** Multinomial logistic regression model for predictors of ED disposition of patients with TBI

Reference (ref): discharged home						
n=3940	Admitted		Died		Others	
	Unadjusted cOR (95% CI)	Adjusted cOR (95% CI)	Unadjusted cOR (95% CI)	Adjusted cOR (95% CI)	Unadjusted cOR (95% CI)	Adjusted cOR (95% CI)
Sex: male	Ref		Ref		Ref	
Sex: female	0.85 (0.70 to 1.04)	0.87 (0.71 to 1.08)	1.01 (0.60 to 1.72)	1.17 (0.65 to 2.10)	0.98 (0.77 to 1.25)	1.00 (0.78 to 1.59)
Age categories: 0–18 years	Ref		Ref		Ref	
19–45	1.15 (0.95 to 1.38)	1.14 (0.94 to 1.39)	1.44 (0.83 to 2.5)	1.56 (0.86 to 2.86)	0.99 (0.79 to 1.25)	0.99 (0.78 to 1.25)
>45	1.22 (0.92 to 1.60)	1.23 (0.93 to 1.64)	1.08 (0.46 to 2.5)	1.33 (0.54 to 3.23)	0.82 (0.57 to 1.17)	0.80 (0.55 to 1.15)
Injury circumstances						
Area of residence: urban	Ref		Ref		Ref	
Rural	0.98 (0.95 to 1.01)	0.98 (0.95 to 1.01)	0.55 (0.37 to 0.83)*	0.54 (0.35 to 0.83)*	0.99 (0.97 to 1.02)	0.99 (0.97 to 1.02)
Place of injuries: home	Ref		Ref		Ref	
Occupational area	1.14 (0.83 to 1.57)	1.16 (0.83 to 1.63)	1.82 (0.74 to 4.5)	2.25 (0.85 to 6.0)	1.38 (0.92 to 2.07)	1.47 (0.97 to 2.24)
Street/Highway	1.14 (0.9 to 1.46)	1.01 (0.76 to 1.34)	1.44 (0.9 to 1.46)	1.63 (0.67 to 3.95)	1.28 (0.93 to 1.75)	1.23 (0.86 to 1.77)
Others	1.03 (0.67 to 1.58)	1.01 (0.64 to 1.57)	0.64 (0.13 to 3.13)	0.50 (0.10 to 2.70)	1.25 (0.73 to 2.13)	1.21 (0.70 to 2.09)
Mode of arrival: private vehicle	Ref		Ref		Ref	
Ambulance	0.97 (0.8 to 1.17)	0.99 (0.83 to 1.21)	1.4 (0.86 to 2.29)	1.54 (0.90 to 2.62)	1.03 (0.81 to 1.32)	1.06 (0.83 to 1.35)
Police vehicle	1.02 (0.85 to 1.22)	1.04 (0.86 to 1.27)	0.99 (0.6 to 1.6)	1.12 (0.64 to 1.98)	1.08 (0.86 to 1.36)	1.11 (0.88 to 1.35)
Motorcycle taxi	1.26 (0.94 to 1.67)	1.32 (0.98 to 1.78)	0.26 (0.06 to 1.13)	0.28 (0.06 to 1.24)	1.29 (0.9 to 1.83)	1.32 (0.92 to 1.89)
Others	0.9 (0.54 to 1.5)	0.91 (0.54 to 1.54)	0.42 (0.05 to 3.22)	0.41 (0.05 to 3.27)	1.25 (0.7 to 2.24)	1.21 (0.67 to 2.20)
Transport time <30 min	Ref		Ref		Ref	
30 min–1 hour	1.11 (0.89 to 1.39)	1.21 (0.96 to 1.52)	0.61 (0.33 to 1.14)	0.68 (0.35–1.32)	1.07 (0.80 to 1.42)	1.15 (0.86 to 1.54)
>1–2 hours	1.04 (0.83 to 1.32)	1.08 (0.85 to 1.38)	0.66 (0.35 to 1.25)	0.65 (0.33 to 1.29)	1.16 (0.87 to 1.56)	1.22 (0.91 to 1.64)
>2–6 hours	1.14 (0.89 to 1.45)	1.15 (0.89 to 1.47)	1.46 (0.83 to 2.58)	1.36 (0.73 to 2.54)	1.29 (0.95 to 1.75)	1.31 (0.96 to 1.78)
>6–24 hours	1.19 (0.87 to 1.63)	1.21 (0.87 to 1.67)	0.88 (0.38 to 2.05)	0.95 (0.39 to 2.32)	1.71 (1.17 to 2.46)*	1.76 (1.21 to 2.57)*
>24 hours	1.15 (0.22 to 1.3.62)	1.23 (0.89 to 1.70)	0.21 (0.05 to 0.92)*	0.26 (0.06 to 1.20)	1.11 (0.74 to 1.65)	1.17 (0.78 to 1.76)
Injury characteristics and severity						
Cause of TBI: RTI	Ref		Ref		Ref	
Falls	0.79 (0.59 to 1.05)	0.81 (0.58 to 1.12)	1.15 (0.57 to 2.32)	1.98 (0.82 to 4.73)	0.96 (0.68 to 1.35)	1.06 (0.72 to 1.57)
Intentional Injuries	0.87 (0.74 to 1.02)	0.83 (0.69 to 0.99)*	0.88 (0.56 to 1.38)	0.91 (0.53 to 1.56)	0.82 (0.67 to 1.00)	0.81 (0.64 to 1.01)
Others	0.81 (0.58 to 1.14)	0.81 (0.57 to 1.17)	1.06 (0.44 to 2.54)	1.46 (0.55 to 3.83)	1.08 (0.72 to 1.60)	1.12 (0.74 to 1.71)
Type of head injury: open	Ref		Ref		Ref	
Closed	0.93 (0.81 to 1.10)	0.91 (0.78 to 1.06)	0.77 (0.51 to 1.15)	0.68 (0.44 to 1.05)	1.02 (0.85 to 1.23)	0.99 (0.82 to 1.20)
Others	1.27 (0.52 to 3.09)	0.98 (0.39 to 2.44)	2.73 (0.56 to 13.26)	1.22 (0.21 to 7.21)	1.38 (0.47 to 4.04)	1.26 (0.43 to 3.73)
**﻿**GCS on arrival: 13–15	Ref		Ref		Ref	
GCS 9–12	1.03 (0.86 to 1.23)	1.48 (1.18 to 1.86)*	1.04 (0.64 to 1.68)	3.04 (1.69 to 5.46)*	0.78 (0.62 to 0.98)*	0.98 (0.74 to 1.31)
GCS ≤8	0.99 (0.81 to 1.21)	1.75 (1.32 to 2.33)*	1.07 (0.63 to 1.82)	9.65 (4.22 to 22.07)*	0.88 (0.68 to 1.12)	1.23 (0.87 to 1.75)
**﻿**Change in GCS: none	Ref		Ref		Ref	
GCS dropped	2.89 (2.37 to 3.52)*	2.92 (2.38 to 3.57)*	31.82 (13.72 to 73.76)*	31.2 (13.23 to 73.56)*	1.54 (1.21 to 1.96)*	1.63 (1.27 to 2.09)*
GCS improved	1.11 (0.94 to 1.32)	0.87 (0.70 to 1.10)	1.5 (0.56 to 4.02)	0.50 (0.17 to 1.49)	0.85 (0.70 to 1.04)	0.86 (0.66 to 1.13)
**﻿**Number of serious injuries: none	Ref		Ref		Ref	
One	0.94 (0.80 to 1.13)	0.94 (0.77 to 1.15)	1.83 (0.98 to 3.40)	1.69 (0.86 to 3.43)	0.77 (0.62 to 0.95)*	0.81 (0.64 to 1.02)
Two or more	1.24 (0.78 to 1.99)	1.17 (0.71 to 1.91)	5.56 (2.11 to 14.62)*	4.52 (1.54 to 13.25)*	0.78 (0.42 to 1.46)	0.74 (0.39 to 1.38)
**﻿**Kampala Trauma Score	0.96 (0.93 to 0.99)*	0.94 (0.90 to 0.98)*	1.00 (0.91 to 1.10)	0.87 (0.78 to 0.98)*	0.95 (0.91 to 0.98)*	0.96 (0.91 to 1.01)
**﻿**Revised Trauma Score	0.98 (0.93 to 1.02)	1.01 (0.95 to 1.06)	0.99 (0.87 to 1.12)	1.06 (0.90 to 1.24)	0.99 (0.94 to 1.05)	0.99 (0.93 to 1.06)

cOR, conditional OR; ED, emergency department; GCS, Glasgow Coma Scale; RTI, road traffic injury; TBI, traumatic brain injury.

*P value <0.05

In the *adjusted multivariable multinomial logistic regression model*, each predictor variable’s odds were compared with the reference category (discharged home) when adjusted for all other predictors.

#### Predictors of admission

Statistically significant predictors with high conditional odds for admission included GCS<13 and drop in GCS. Meanwhile intentional injuries and a higher KTS had significantly lower conditional odds for admission when compared with the reference (discharged home).

#### Predictors of death in the ED

Statistically significant higher conditional odds of death were observed for GCS<13 on arrival, a drop in GCS, and two or more serious injuries, after adjusting for other variables. Significantly lower conditional odds of death were observed for those from rural areas and those who arrived after 24 hours of injury, when adjusted for other predictor variables. Higher or favorable KTS had statistically significant lower conditional odds of death observed in the adjusted model.

#### Predictors of others

For those who were in the “others” category, after adjusting for other variables, had an arrival time between 6 and 24 hours, and a drop in GCS during ED stay had significantly higher conditional odds when compared with discharge home. The effects of the number of injuries and KTS disappeared in the adjusted model. Sex, age, area of residence, place of injuries, transport time, cause of TBI, initial GCS, number of injuries, and KTS and RTS were not statistically significant predictors of “Others” ED disposition in an adjusted multinomial regression model.

The *final model based on the lowest AIC* had only four predictor variables, that is, area of residence, KTS, number of serious injuries, and change in GCS (table 4). Rural area of residence was associated with significantly lower odds of death when compared with the “discharged home” category. Having a high KTS had lower conditional odds for being in the “Admission” category. A drop in GCS was associated with statistically significantly higher conditional odds for all ED disposition categories as compared with those who were discharged home. Having one serious injury was associated with lower odds of being in the “others” category when compared with those who were sent home, whereas two or more injuries were associated with high conditional odds for ED death as compared with being discharged, although this association was not statistically significant.

**Table 4:** Final model: determinants of emergency department discharge disposition of patients with TBI in Mulago Hospital

	Reference: discharged home		
	Admitted	Died	Others
	Adjusted cOR (95% CI)		
Area of residence (urban)	Ref		
Rural	0.98 (0.95 to 1.01)	0.50 (0.33 to 0.75)*	0.99 (0.97 to 1.02)
KTS	0.96 (0.93 to 0.99)*	1.01 (0.91 to 1.11)	0.97 (0.92 to 1.01)
Number of serious injuries: none	Ref		
One	0.92 (0.76 to 1.12)	1.39 (0.71 to 2.68)	0.79 (0.63 to 0.99)*
Two or more	1.06 (0.65 to 1.72)	2.70 (0.97 to 7.51)	0.73 (0.39 to 1.37)
Change in GCS: none	Ref		
GCS dropped	2.97 (2.43 to 3.63)*	31.53 (13.55 to 73.39)*	1.61 (1.26 to 2.06)*
GCS improved	1.18 (0.99 to 1.41)	1.50 (0.55 to 4.07)	0.91 (0.73 to 1.12)

*P<0.05.

ED, emergency department; GCS, glasgow coma scale; KTS, Kampala trauma score; cOR, conditional OR; ref, reference.

## Discussion

Our study is the first study, to our knowledge, that uses TBI registry data to predict ED disposition, and hence highlights several important issues related to TBI care in Uganda. First, the study demonstrated a high burden of patients with TBI presenting to the ED, of both primary and referred TBI cases. Since Mulago is the only public sector tertiary-care hospital caring for neurosurgical patients and national trauma referral center, this finding is not surprising. The majority of patients were young men, equally representing urban and rural areas, as reported in a previous study from the same hospital.^27^ A disproportionately high number of injuries were reported from roads, and in addition to RTIs intentional injuries were an important cause of TBIs. It is important to note that the proportion of intentional injury is higher than in previous reports.^15^ Since about 70% of patients had mild head injury based on GCS, with no other serious injury, it is not surprising that about a third were discharged home.

Delay in reaching the hospital was commonly observed, although it did not appear to have a statistically significant impact on ED discharge disposition. In fact, comparatively lower odds of death among those who survived the first 24 hours demonstrated that their survival was better owed to the stability of clinical condition, and it was also likely that they would be discharged home after receiving care in the ED. Prehospital delays in critically ill patients could potentially result in worsening of GCS either due to primary TBI or due to secondary brain injury from hypotension, and compromised airway, and hence it is possible that some patients might not have reached Mulago Hospital because of improper assessment, delays in recognizing warning signs, or unavailability of transport.

The lack of effective emergency medical systems in Uganda presents a barrier in early resuscitative measures that could potentially improve TBI outcomes.^28 29^ This was manifested by delays in presentation, non-ambulance prehospital transport, and interfacility transfers in the subjects of this study. A possible explanation of lower conditional odds for death among TBI victims from rural areas could be because of a slightly lower proportion of severe head injuries from rural areas compared with patients from urban areas (47% vs 52%), although this association was not statistically significant (p=0.65). It could also mean that patients with severe TBIs in rural areas die in the prehospital space and those that do make it to the tertiary hospital had minor or stable injuries to begin with.

Our study also identifies a few important issues related to TBI care in Mulago Hospital. The study demonstrated that change in GCS was the single most important predictor of ED disposition in patients with TBI. Although the timing of this second GCS recording ranged between 2 and 4 hours, it is important to note that a third of all patients presenting with TBI suffered a drop in GCS during ED stay, and this was a strong predictor of inpatient admission and for those who died in the ED. This fact highlights the importance of early identification of patients with TBI at risk (such as those with moderate or severe head injury and multisystem involvement), and underscores the value of early and aggressive resuscitative measures in TBIs.^14 30^ This observation is supported by the fact that 60% (n=65) of those who died in the ED were categorized as mild head injury (GCS between 13 and 15 patients on the basis of the first GCS recording) and perhaps were not prioritized to receive early multidisciplinary management. This finding should lead to improved assessment and resuscitation guidelines, targeted efforts to identify patients with a potential for episodic or sustained hypotension and airway compromise, and systems-based measures to promptly provide definitive care.^30 31^ Even in resources-poor settings, complete vital signs especially GCS must be monitored at short, regular intervals in all ED trauma patients, especially during resuscitation.

RTS and KTS were integrated as main injury severity measures in this registry. Unlike RTS which indicates only physiologic status, KTS is a composite injury severity measure (it uses vital signs, age, and number of injuries to calculate injury severity) that has been validated in other low-resource settings.^32^ KTS was initially developed to support ED decision making, as well as predict admission or death.^33^ In our study, higher KTS showed less adjusted odds of admission, but had no significant association with death. Similarly, RTS did not predict ED disposition, and hence in case of TBIs the best predictor of admission or death remained the initial GCS and change in GCS. This finding also carries significant importance in low-resource settings. The fact that many patients face delays in imaging and other diagnostics, simple bedside monitoring of GCS, especially conscious level, could help ED care providers in prioritizing care and mobilizing resources to avoid catastrophic outcomes.

About 15% of patients were still waiting for final disposition even after 24 hours of ED stay. This was often due to delays in investigations or critical resources such as ICU availability. The logistical and financial barriers to have early CT scans and ICU care have been previously identified in Mulago Hospital.^31^ With such barriers, patients sometimes forego critical investigations and treatment. Establishing more public sector neurosurgical services will definitely take the pressure off Mulago Hospital and provide more options to the patients. It has been previously demonstrated that high-volume LMIC trauma centers are chronically overcrowded to the point of diminished effectiveness.^34^ This study provides the evidence to improve the quality of trauma care in Ugandan tertiary-care hospitals, as well as helps policy makers and healthcare planners to mobilize material resources, establish guidelines, and decentralize trauma care to district hospitals.

### Limitations

Only those patients who were able to give informed consent, or for those whose parents or next of kin were available to provide assent or permission, were included in this registry. Some patients with severe head injury, those with depressed consciousness due to multisystem involvement, or early deaths might have been excluded from the registry, as they were unable to provide consent. This might have resulted in a relative preponderance of mild and moderate TBI cases. This study is based on a 15-month data; although there might be little effect of seasonality on TBI incidence, this cannot be entirely excluded. Patients who were brought in dead or left without being seen by a healthcare provider are also not included in the registry. Some variables such as diagnostic tests or treatment details that could have some impact on the ED disposition were not included in the analysis.

## Conclusion

TBI registry implemented in a tertiary-care hospital of Kampala, Uganda, can be used to quantify the hospital burden, clinical care issues, and patient outcome. The ORs for ED disposition are differentially affected by injury characteristics and are largely dependent on injury severity and change in GCS during ED stay. The single most important bedside examination that could predict disposition from ED of patients with TBI is serial GCS monitoring, which should be implemented even in the most resource-constrained settings.
